# Clinical utility of 1:16 serum dilution as a predictor of response to therapeutic plasma exchange for HLA antibody-mediated rejection treatment and overall survival in lung transplant recipients: A two center study

**DOI:** 10.1016/j.jhlto.2025.100302

**Published:** 2025-05-29

**Authors:** Mohamed Elrefaei, Tathagat Narula, Francisco Alvarez, Elizabeth A. Godbey, Jasmine Kendrick, Gerard Criner, Francis C. Cordova, Norihisa Shigemura, Yoshiya Toyoda, Olga Timofeeva

**Affiliations:** a Department of Laboratory Medicine and Pathology, Mayo Clinic, Jacksonville, FL; b Division of Lung Failure and Transplant 2, Mayo Clinic, Jacksonville, FL; c Department of Thoracic Medicine and Surgery, Lewis Katz School of Medicine, Temple University, Philadelphia, PA; d Department of Surgery, Lewis Katz School of Medicine, Temple University, Philadelphia, PA; e Department of Pathology and Laboratory Medicine, David Geffen School of Medicine at UCLA, Los Angeles, CA

**Keywords:** Lung transplant, Rejection, HLA antibodies, Serum dilution, Plasma exchange

## Abstract

**Purpose:**

Antibody-Mediated Rejection (AMR) due to HLA donor-specific antibodies (DSA) is associated with poor outcomes in lung transplant recipients (LTR). AMR treatment using therapeutic plasma exchange (TPE) improves clinical outcomes in LTR. The objective of this study was to assess the clinical utility of 1:16 serum dilution HLA antibody test results as a predictor of response to TPE for de novo DSA (dnDSA) levels and AMR treatment in LTR.

**Methods:**

A retrospective analysis of 32 LTR diagnosed with AMR due to dnDSA and treated with TPE was performed at Mayo Clinic (n = 18) and Temple University Hospital (n = 14). HLA antibodies were detected by Luminex single antigen beads assay. Mean Fluorescence Intensity (MFI) levels were measured in undiluted and 1:16 diluted sera before the 1st and after the 5th TPE session. Statistical analysis was performed using GraphPad PRISM software.

**Results:**

Of 32 patients, 14 and 18 patients were diagnosed with early (< 3 months post-transplant) and late (6 months – 3 years post-transplant) AMR respectively. All patients, except one, had HLA Class II dnDSA (97%). In addition, 9/14 (64.2%) and 3/18 (16.6%) of LTR with early and late AMR respectively had HLA class I dnDSA. The MFI for all positive dnDSA in 1:16 diluted sera collected before 1st TPE demonstrated a significant correlation with MFI in undiluted sera collected after 5th TPE in both early (R^2^ = 0.8786) and late (R^2^ = 0.9045) AMR post-transplant. In addition, MFI in 1:16 diluted pre TPE sera correlated with better overall LTR survival following TPE (p = 0.001).

**Conclusion:**

The MFI of 1:16 serum dilution before 1st TPE may be utilized as a surrogate to predict response to TPE for AMR treatment and overall survival in LTR.

## Introduction

The long-term survival after lung transplant remains low with a 5–year survival rate of approximately 55%.^1^ Human leukocyte antigens (HLA) donor-specific antibodies (DSA) are associated with antibody-mediated rejection (AMR), chronic lung allograft dysfunction (CLAD), and decreased survival in lung transplant recipients (LTR).^2^, ^3^, ^4^, ^5^ Early AMR may be mediated by activation of memory response post-transplant and production of DSA in LTR. In contrast, late AMR caused by de novo DSA (dnDSA) production likely occurs through differentiation of naïve B cells into plasma cells that requires activation of indirect alloimmune pathway.^6^, ^7^ LTR who clear HLA DSA following immunomodulatory therapy have better survival than those with persistent HLA DSA and similar AMR incidence as those who do not develop HLA DSA.^3^, ^8^, ^9^, ^10^ Preemptive early treatment of dnDSA may reduce the subsequent risk of CLAD and death in LTR.^11^ In this study, all DSA that were not detected pre transplant will be called dnDSA.

While there is no standardized optimal AMR treatment, several therapeutic strategies including therapeutic plasma exchange (TPE) to quickly deplete circulating HLA DSA,^12^, ^13^ proteasome inhibitor bortezomib, high dose intravenous immunoglobulin, and rituximab are used for AMR treatment in LTR.^14^, ^15^ Previous studies have demonstrated that TPE can reduce total HLA antibody levels compared to pre TPE levels.^16^, ^17^, ^18^, ^19^ However, there are no clinical predictors or biomarkers for predicting if TPE may decrease HLA DSA levels and reverse AMR.

Solid-phase single antigen bead HLA antibody test is used for dnDSA detection and monitoring response to DSA reduction therapy based on the mean fluorescence intensity (MFI) value.^20^, ^21^ However, dnDSA MFI alone may not predict an effective response to TPE for AMR treatment.^22^ We previously demonstrated that 1:16 serum dilution HLA antibody test may be used as an additional biomarker to predict response to TPE for a particular antibody in heart and lung transplant.^22^, ^23^ However, there was only weak or no positive correlation between MFI values in undiluted pre-TPE compared to post-TPE MFI.^22^, ^24^ Our objective in this two-center study was to assess the clinical utility of 1:16 serum dilution HLA antibody test results as a surrogate to predict response to TPE for dnDSA production, AMR treatment, and overall survival in LTR.

## Methods

### Study subjects and source of samples

A retrospective analysis of 32 LTR diagnosed with AMR due to dnDSA and treated with TPE was performed at Mayo Clinic in Florida (MCF; n = 18) and Temple University Hospital (TUH; n = 14). All LTR were assessed and monitored pre-and post-transplant for the presence of HLA antibodies per institutional protocol. The study was approved by the MCF and TUH Institutional Review Board and all study participants provided informed written consent pre transplant. Inclusion criteria included all primary and re-transplant LTR that were > 18 years old, irrespective of gender and race. Recipients undergoing multi-organ transplantation were excluded from the study. Demographic, clinical information, and lab test results of the study participants were reviewed as previously described.^25^

### HLA antibody detection

Serum samples were treated with EDTA to avoid non-specific inhibition and tested for IgG HLA class I and II antibodies using the LABScreen single antigen beads (One Lambda-ThermoFisher, Inc.) at MCF and TUH as previously described.^26^ The presence of HLA DSA against HLA-A, -B, -C, -DRβ, -DQα/β, and -DPβ antigens was determined. Positive HLA DSA were defined as having a MFI of more than 1000. No false positive or non-specific test results were included in the study analysis. No HLA DSA with MFI < 1000 were detected in undiluted serum pre 1st TPE session. The MFI levels were measured before the 1st and one day after the 5th TPE session using undiluted and 1:16 diluted sera in PBS. Different lots of the HLA antibody test were used in each center and demonstrated no significant differences in results between lots. HLA antibody test results following more than 5 TPE sessions were not included in the study analysis.

### TPE for AMR treatment

TPE was performed using the Spectra Optia (Terumo BCT, Lakewood, CO) with replacement fluid of 5% albumin and/or plasma (replacement fluid dictated by patient-specific factors). TPE was performed daily at MCF over 4 days in 2 LTR and 5 days in 16 LTR. TPE was performed every other day at TUH as previously described.^22^

### Induction immunosuppression

At MCF 83% and 17% of LTR received a single dose of rabbit Antithymocyte Globulin or Alemtuzumab for induction immunosuppression respectively. At TUH 60% and 40% of LTR received Basiliximab or Alemtuzumab respectively. All LTR in the study were treated post-transplant with a standard immunosuppressive regimen incorporating systemic corticosteroids, calcineurin inhibitor, and an antimetabolite.

### Statistical analysis

Statistical analysis including log-linear regression was performed using GraphPad PRISM software (10.3.1; GraphPad). Statistical significance was defined as p < 0.05. One-way ANOVA was used when comparing three groups. Survival curves were plotted using the Kaplan–Meier method and groups were compared by log-rank testing. For survival analyses, we calculated the percent reduction of dnDSA with the highest MFI value. Previous studies have demonstrated a significant correlation between HLA DSA with high MFI values and flow crossmatch results.^27^

## Results

### Correlation of HLA antibody test between two centers

To establish that dilution results are concordant between different centers, serum samples from 3 LTR were tested in parallel for HLA antibodies at MCF (center 1) and TUH (center 2). We observed a significant correlation between the 2 centers in HLA class I and II antibody MFI levels of undiluted (R^2^ = 0.9862 and R^2^ = 0.9851), 1: 4 dilution (R^2^ = 0.9934 and R^2^ = 0.9903), and 1: 16 dilution (R^2^ = 0.9938 and R^2^ = 0.9932) sera aliquots (supplemental Figure 1). These results demonstrate that dilution studies at different centers provided comparable results, and, therefore, can be used for inter-center comparisons. We previously assessed different serum dilutions and demonstrated that 1:16 serum dilution HLA antibody test may be used as an additional biomarker to predict response to TPE.^22^, ^23^, ^24^

### Detection dnDSA in LTR with early and late AMR

A total of 32 LTR diagnosed with AMR due to HLA dnDSA and treated with TPE at MCF (n = 18) and TUH (n = 14) were included in the study. The diagnosis of AMR was based on ISHLT consensus report on AMR of the lung.^28^ All LTR were diagnosed with AMR based on presence of HLA DSA, symptomatic or asymptomatic measurable allograft dysfunction, and exclusion of other causes of allograft dysfunction such as infection, airway pathology, cellular rejection, volume overload). No LTR with histology proven AMR/positive C4d staining on pathology specimens was included.

All LTR had a negative flow cytometry crossmatch on the day of transplant. Of 32 patients, 14 and 18 LTR were diagnosed with early (< 3 months post-transplant) and late (> 6 months - 5 years post-transplant) AMR respectively. A total of 14 and 57 HLA class I and II dnDSA respectively were detected in undiluted serum samples pre 1st TPE (Table 1). In addition, a total of 9 and 43 HLA class I and II dnDSA respectively were detected in undiluted serum samples post 5th TPE. The most prevalent dnDSA specificity was DQB1 and was detected in 39 and 35 undiluted serum samples pre 1st and post 5th TPE respectively.

**Table 1:** Prevalence of HLA Class I and II dnDSA a

dnDSA	Pre 1st TPE	Post 5th TPE	Total
HLA class I	14	9	23
A	8	5	13
B	6	4	10
C	0	0	0
HLA class II	57	43	100
DRB1	7	4	11
DRB3/4/5	3	2	5
DQB1	39	35	74
DPB	8	2	10

a Number of HLA class I and II dnDSA detected in undiluted serum samples pre 1st TPE and post 5th TPE

**Figure 1:**
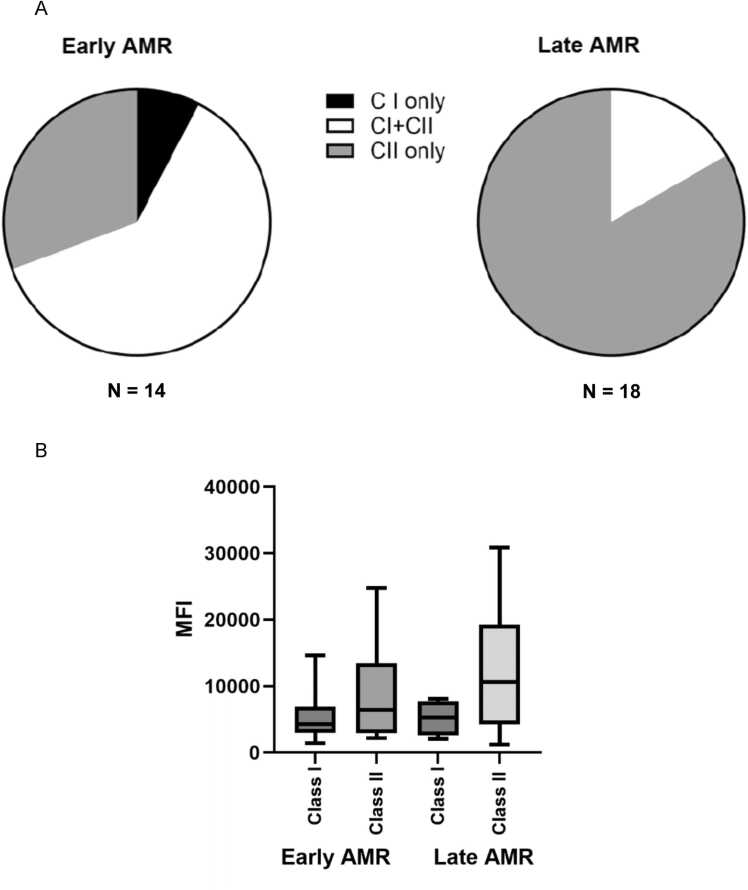
Detection dnDSA in LTR with early and late AMR. (A) distribution and (B) MFI of HLA class I or II dnDSA in early and late AMR.

All patients, except one, had HLA Class II dnDSA (97%). In addition, 9/14 (64.2%) and 3/18 (16.6%) of LTR with early and late AMR respectively had HLA class I dnDSA (Figure 1A). Differences in MFI between HLA class I versus II antibodies in early (p = 0.2) and late (p = 0.1) AMR were not statistically significant. (Figure 1B). In addition, there were no significant differences in MFI of HLA class I (p = 0.9) and II (p = 0.1) antibodies between early versus late AMR.

### Correlation of dnDSA MFI between 1: 16 serum dilution and undiluted serum following TPE

**Figure 2:**
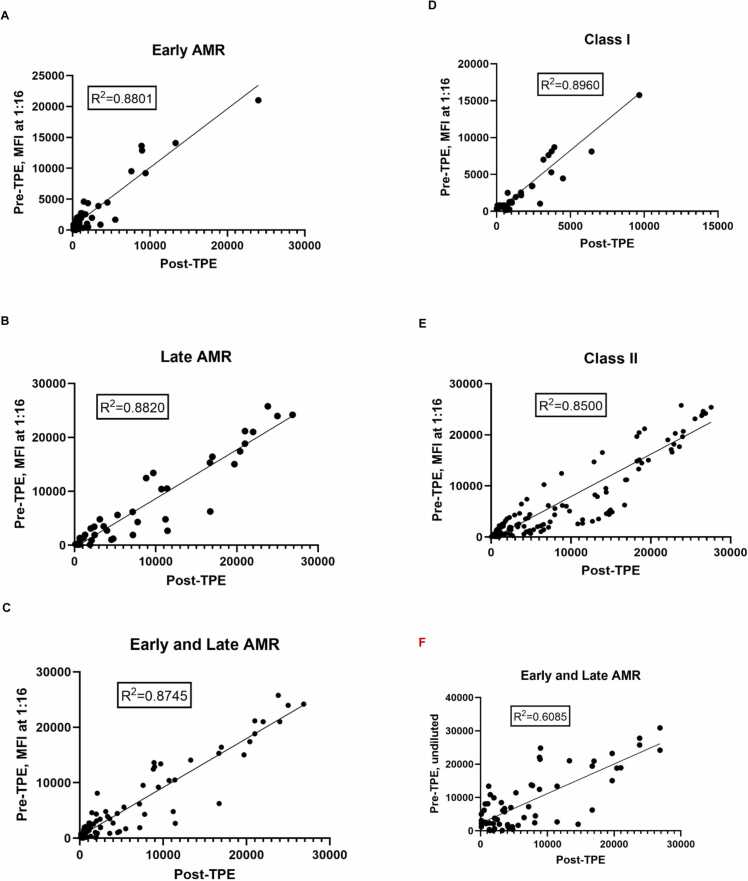
MFI levels of HLA antibodies were measured before the 1st and after the 5th TPE session using undiluted and 1:16 diluted sera in (A) early (< 3 months post-transplant), (B) late (> 6 months post-transplant), (C) combined early and late AMR, (D) all class I, and (E) all class II dnDSA. (F) correlation between dnDSA MFI from combined early and late AMR in undiluted pre-TPE compared to post-TPE serum.

MFI levels of dnDSA were measured before the 1st and after the 5th TPE session using undiluted and 1:16 diluted sera. The MFI for all positive dnDSA in 1:16 diluted sera collected before 1st TPE demonstrated a significant correlation with MFI in undiluted sera collected after 5th TPE in early (R^2^ = 0.8801; Figure 2A), late (R^2^ = 0.8820; Figure 2B), and combined early and late AMR (R^2^ = 0.8745; Figure 2C). A similar significant correlation was also observed in all Class I (R^2^ = 0.8960; Figure 2D) and Class II (R^2^ = 0.8500; Figure 2E) dnDSA. However, the correlation between dnDSA MFI from combined early and late AMR in undiluted pre-TPE compared to post-TPE serum was less significant (R^2^ = 0.6085; Figure 2F).

### Correlation between dnDSA MFI in 1:16 serum dilution and overall LTR survival following TPE

**Figure 3:**
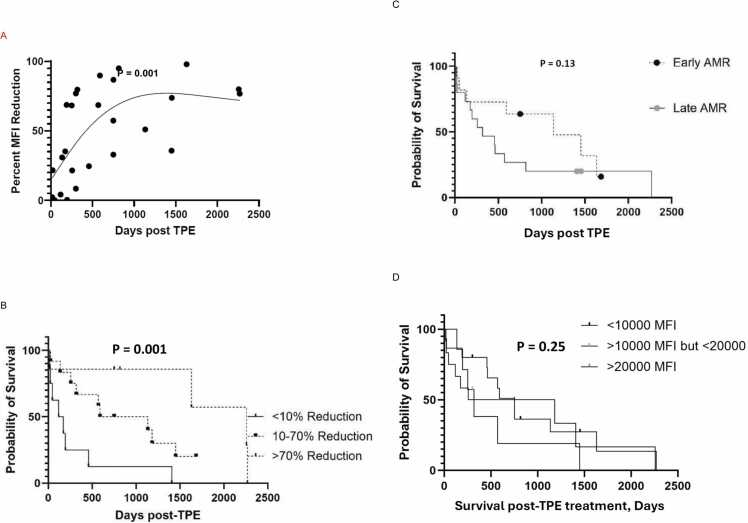
Relationship between dnDSA and overall patient survival. (A) a restricted cubic spline analysis of dnDSA percent MFI reduction after the 5th TPE session. (B) correlation between dnDSA MFI in 1:16 serum dilution and overall LTR survival following TPE based on percent of MFI decrease in 1:16 diluted serum. All patients were divided into three groups (with <10% decrease, 10 – 70% decrease, and >70% decrease). (C) difference in overall survival of LTR diagnosed with early compared to late AMR. (D) correlation between dnDSA MFI in undiluted sera before the 1st TPE and overall LTR survival

We assessed the relationship between the percent reduction in dnDSA MFI in 1:16 diluted sera and overall LTR survival following 5th TPE session using a restricted cubic spline analysis (Figure 3A). Based on this analysis, all LTR were divided into three groups based on percent of MFI decrease of dnDSA in 1:16 diluted serum, (<10% decrease, 10 – 70% decrease, and >70% decrease). Reduction in MFI of dnDSA in 1:16 diluted sera correlated with better overall LTR survival following TPE (Figure 3B; p = 0.001). LTR whose dnDSA did not decrease in 1:16 diluted sera had worst overall survival, while LTR who demonstrated a partial response to treatment had improved survival. The difference in overall survival of LTR diagnosed with early compared to late AMR was not statistically significant (Figure 3C; p = 0.13). In addition, the correlation between dnDSA MFI in undiluted sera before the 1st TPE and overall LTR survival was not statistically significant (Figure 3D; p = 0.25) which suggests that pre-TPE dnDSA MFI do not predict post-TPE LTR survival.

## Discussion

This is the first 2 center study to report a significant correlation between 1:16 serum dilution HLA antibody test results and response to TPE for dnDSA levels, AMR treatment, and overall survival in LTR. In the last few years numerous investigations highlighted the deleterious effects of dnDSA in antibody-mediated allograft damage in LTR and increased mortality.^2^, ^3^, ^25^, ^29^, ^30^, ^31^, ^32^, ^33^, ^34^, ^35^ Detection of dnDSA within one month post lung transplant was shown to be associated with worse survival.^3^, ^32^ Persistent dnDSA was recently shown to pose a significant risk on allograft function and LTR survival compared to transient dnDSA.^36^ Results of our current study leads us to postulate that the ability to better predict response to TPE may allow for early identification of LTR that will require more aggressive AMR regiment treatment leading to better transplant outcome. Diagnosis of AMR in LTR based on clinical, pathologic, serologic, and immunological criteria is challenging. The degree of certainty in diagnosis translates to classifying LTR into subsets of definite, probable, and possible AMR resulting in varying LTR presentations ranging from asymptomatic with subclinical abnormalities to frank respiratory failure.^28^ However, there is no consensus on who and when to treat, optimal treatment strategy, and duration of treatment. The knowledge gap extends to our inability to prognosticate response to available therapeutics for optimal outcomes for AMR in LTR.

The objectives of treating AMR include reducing circulating HLA DSA levels, suppressing additional HLA DSA development, and preventing lung injury. TPE is commonly utilized as a first-line treatment and may result in a rapid decrease in circulating HLA DSA levels. However, the response to TPE in LTR with AMR is inconsistent and ranges from complete clearance of HLA DSA with clinical stabilization and improvement to almost no change in HLA DSA levels and rapid clinical decline.^37^, ^38^, ^39^ Recognizing that successful HLA DSA depletion is critical for a favorable clinical response; predictive models have inherent clinical value before subjecting the patient to multiple rounds of an invasive intervention like TPE. Our study builds on prior work highlighting the predictive value of undiluted and diluted sera on HLA DSA levels and AMR prognosis following TPE.^22^ We observed a significant correlation between HLA DSA levels in 1:16 serum dilution and response to TPE and overall survival in LTR. The significant differences between survival based on the percent of MFI decrease in 1:16 diluted serum may allow for prognostication at the onset of treatment. This is especially valuable in LTR treated for HLA DSA without allograft dysfunction because traditional response metrics may be unavailable. It is critical that we provide LTR with a gauge of the expected response from standard therapeutics, allowing for reasonable expectations and care planning for the future.

Findings from this study raise relevant questions that need clinical consideration and further investigation. Should LTR who do not show adequate reduction in HLA DSA with 1:16 serum dilution be offered TPE ? or instead be offered more aggressive therapeutics including a higher number of TPE sessions? The limited available literature suggests that an aggressive protocol incorporating 8 TPE sessions and Bortezomib may significantly decrease HLA DSA and improve overall survival in LTR that did not demonstrate significantly lower HLA DSA levels in 1:16 serum dilution.^22^ Alternatively, Carfilzomib and Daratumumab may be administered as alternative therapies for AMR treatment.^40^ Incorporating prognostic markers of response to potent immunomodulatory and immunosuppressive therapies in research endeavors is paramount for the early identification of LTR that will require more aggressive AMR regiment treatment beyond TPE and prevent undue LTR exposure to potentially toxic therapeutics. The results of our current study are a step in this direction.

## Limitations

The primary limitation of our study is the small number of patients and a relatively short follow-up period post TPE may have limited the ability to identify significant differences in long-term outcomes. In addition, in-vitro immunologic data often do not adequately address and mirror the complexity of in-vivo immune processes. However, our study underlines the need for a larger randomized prospective study to elucidate the long-term benefits on allograft function from early identification of LTR that will require more aggressive AMR treatment. In addition, a more balanced study cohort is needed to validate our initial findings and optimize HLA antibody testing strategies to minimize the risk of allograft injury in LTR.

## Conclusion

AMR is a major cause of allograft failure in LTR highlighting the importance of AMR prevention and treatment.^41^ The MFI of 1:16 serum dilution pre TPE may be utilized as a surrogate to predict response to TPE for AMR treatment and overall survival in LTR. Predicting response to TPE may allow for early identification of LTR that will require more aggressive AMR regiment beyond TPE leading to better transplant outcome. The correlation between the1:16 serum dilution HLA antibody test results and lung allograft function tests should be examined in future studies.

## Funding source

None.

## Authors disclosures

None.

## Declaration of Competing Interest

The authors declare that they have no known competing financial interests or personal relationships that could have appeared to influence the work reported in this paper.
