# Supplementary material for: Clinical utility of 1:16 serum dilution as a predictor of response to therapeutic plasma exchange for HLA antibody-mediated rejection treatment and overall survival in lung transplant recipients: A two center study
Source: JHLT Open. 2025 May 29;9:100302. doi: 10.1016/j.jhlto.2025.100302 (PMC12221467; doi:10.1016/j.jhlto.2025.100302)

Supplemental figure 1. Serum samples from 3 LTR were simultaneously tested for HLA class I (A) and II (B) antibodies at Mayo Clinic in Florida (center 1) and Temple University Hospital (center 2). correlation between the 2 centers in the HLA class I and II antibody MFI levels of undiluted, 1 : 4 dilution, and 1 : 16 dilution sera aliquots.

Supplemental figure 1


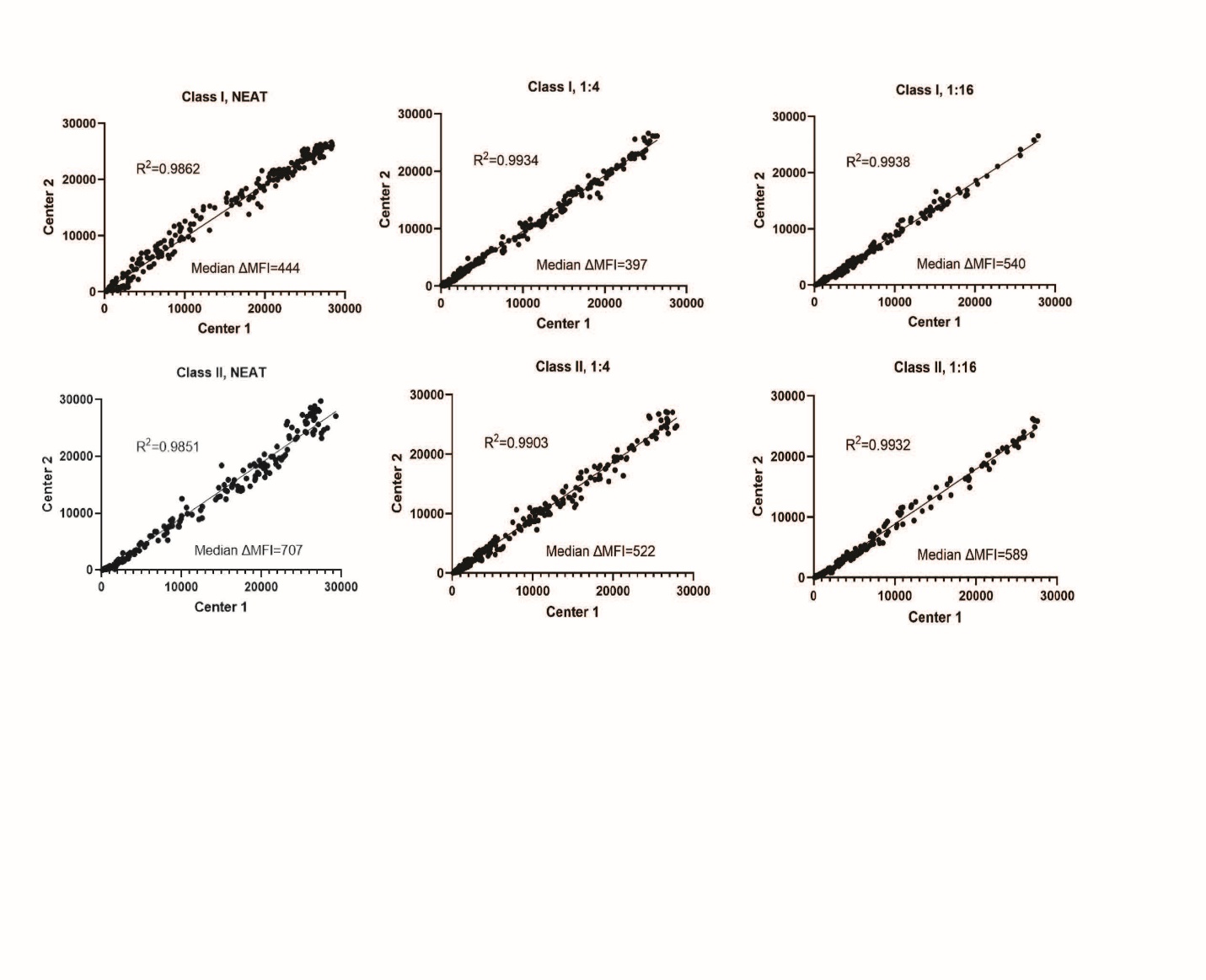

Supplement: Supplementary file 1 — Supplemental material [file mmc1.docx]
